# Early intervention in Alzheimer’s disease: a health economic study of the effects of diagnostic timing

**DOI:** 10.1186/1471-2377-14-101

**Published:** 2014-05-07

**Authors:** Jennifer H Barnett, Lily Lewis, Andrew D Blackwell, Matthew Taylor

**Affiliations:** 1 Cambridge Cognition, 9 Tunbridge Court, Bottisham, CB25 9TU Cambridge, UK; 2 Department of Psychiatry, University of Cambridge, Cambridge, UK; 3 York Health Economics Consortium, University of York, York, UK

**Keywords:** Alzheimer’s disease, Dementia, Diagnosis, Treatment, Early intervention, Health economics, Cost-effectiveness, Cholinesterase inhibitors

## Abstract

**Background:**

Intervention and treatment in Alzheimer’s disease dementia (AD-dementia) can be cost effective but the majority of patients are not diagnosed in a timely manner. Technology is now available that can enable the earlier detection of cognitive loss associated with incipient dementia, offering the potential for earlier intervention in the UK health care system. This study aimed to determine to what extent the timing of an intervention affects its cost-effectiveness.

**Methods:**

Using published data describing cognitive decline in the years prior to an AD diagnosis, we modelled the effects on healthcare costs and quality-adjusted life years of hypothetical symptomatic and disease-modifying interventions. Early and standard interventions were assumed to have equal clinical effects, but the early intervention could be applied up to eight years prior to standard diagnosis.

**Results:**

A symptomatic treatment which immediately improved cognition by one MMSE point and reduced in efficacy over three years, would produce a maximum net benefit when applied at the earliest timepoint considered, i.e. eight years prior to standard diagnosis. In this scenario, the net benefit was reduced by around 17% for every year that intervention was delayed. In contrast, for a disease-modifying intervention which halted cognitive decline for one year, economic benefits would peak when treatment effects were applied two years prior to standard diagnosis. In these models, the maximum net benefit of the disease modifying intervention was fifteen times larger than that of the symptomatic treatment.

**Conclusion:**

Timeliness of intervention is likely to have an important impact on the cost-effectiveness of both current and future treatments. Healthcare policy should aim to optimise the timing of AD-dementia diagnosis, which is likely to necessitate detecting and treating patients several years prior to current clinical practice.

## Background

Dementia is estimated to affect 36 million people worldwide, costing more than US $600 billion a year [1]. The ageing demographics of both developed and developing nations mean that its prevalence will double over the next 20 years [2]. Alzheimer’s disease dementia (AD-dementia) is the most common form, estimated to account for 60 to 80% of cases [3]. An additional 5-42% of older adults [4] meet criteria for mild cognitive impairment (MCI), a lesser degree of impairment than dementia, which allows them to continue to function independently [5].

The current model of Alzheimer’s disease is of a pathological process which starts in mid-life but remains undetected until it causes dementia, at which time a clinical diagnosis of AD-dementia can be made. Recent revisions to diagnostic criteria define three stages of Alzheimer’s disease, namely Alzheimer’s disease dementia [6], MCI due to AD (MCI-AD) [7], and preclinical AD [8]. Broadly speaking, the MCI and dementia stages of AD can be determined by a combination of clinical assessment and biomarkers, while detection of preclinical AD, characterised by the absence of overt symptoms, requires brain-related assays of disease progression [9-11]. Within the MCI population a significant proportion have symptoms due to underlying AD pathology (MCI-AD [7]). Episodic memory impairment is typically a common cognitive marker in those with MCI who progress to AD-dementia, and each year around 15% of individuals with amnestic (memory-impaired) MCI are diagnosed with AD-dementia [5,9].

Technology now exists that can enable the earlier detection of cognitive loss associated with incipient dementia, offering the potential for earlier intervention in the UK health care system ([12,13]). Timely detection and intervention in AD-dementia can be cost-effective because even though current treatments have limited efficacy, they nonetheless improve symptoms enough to reduce healthcare costs and keep patients living in the community for longer. Although the debate over cost-effectiveness of current treatment is controversial, some economic studies have estimated that timely treatment is cost-effective [14]. A UK study based on 2007 costs estimated that over ten years, timely detection and treatment produced savings of £3600 (US $5508) in direct costs and an additional £4150 ($6350) in indirect costs (caregiver time) per patient [15]. Similar cost-effectiveness has been established for other healthcare systems, including the US [16]. Despite this, in the UK as in other countries, the majority of people with AD-dementia do not have a diagnosis, with up to three quarters undiagnosed in some regions [17-19].

Improved public awareness of AD-dementia is driving older adults who experience memory problems to seek help at an increasingly early stage. At presentation, many of these individuals may meet criteria for MCI-AD for which, as yet, there are no licensed treatments [20]. There is considerable debate within the clinical and public health communities as to the stage at which it is most appropriate to assess and treat people who may have, or be worried about, the early signs of AD-dementia. There is generally little economic evidence about pre-dementia stages of AD (MCI-AD) [21], but it is likely that resource use increases considerably in the years preceding an AD diagnosis [22].

One key factor determining when detection and diagnosis programmes should be targeted is understanding at what stage in disease the available interventions are most clinically effective. Unfortunately, there is little data on the size of clinical effect at different stages of disease for even the most common treatments such as cholinesterase inhibitors. It is generally thought that these interventions are more effective when used before widespread pathological change has occurred [23-25], but there is little evidence that they are effective in pre-dementia stages such as MCI-AD [20,26].

A second major factor is economic: the extent to which healthcare costs and quality of life are changed by intervening at different points in the disease course. In this study we aimed to assess the economic effects of intervening in AD-dementia up to 9 years earlier than that which currently occurs. In the absence of detailed data comparing the efficacy of treatments at different stages, we concentrated only on the economic impact of applying (equally effective) interventions at different points in the disease course. Because few treatments are currently available, we modelled the effects of two hypothetical interventions: one a modestly-effective symptomatic treatment, the other a hypothetical disease-modifying treatment (DMT) that halted cognitive decline for a short period. These two scenarios were chosen as simple and relatively conservative examples of the type of intervention that might plausibly achieve widespread adoption within a healthcare system such as the UK NHS or US Medicare. In this way we aimed to quantify the extent to which the potential costs and savings from an intervention for AD-dementia would be impacted by the time of intervention.

## Methods

### Data and cost assumptions

Our model was based on the reported natural history of cognitive decline in the nine years preceding a diagnosis of Alzheimer’s disease (AD-dementia) [27] in a longitudinal population-based study in the southwest of France, the Paquid cohort [28]. In this study, cognitive function was monitored in 1285 individuals who were initially assessed as not being demented, including 215 who were subsequently diagnosed with dementia due to Alzheimer’s disease. These data are therefore a description of the average cognitive decline experienced during pre-diagnosis AD. With a mean Mini Mental State Examination (MMSE) [29] score of around 26 at the start of this time period and 18 at the time of diagnosis, they reflect not the ideal of timely diagnosis but the clinical reality that many patients receive a diagnosis many years after the onset of AD-dementia.

We implemented a cohort model where all individuals follow either the standard pattern of decline or received early intervention as described below. The cost of institutional care, and the relationship between MMSE and direct non-institutional healthcare costs were reported by Knapp & Prince in 2007 [30] based on the UK National Health Service (NHS). These were inflated to 2011 costs using the PSSRU Hospital and Community Health Services Index [31]. Costs and benefits were discounted at a rate of 3.5% per year. The relationship between probability of institutionalisation and MMSE score was derived following Getsios et al’s methodology [15], based on the national prevalence of AD-dementia severities and the known severity of patients in institutional care [32].

### Baseline calculations

Our baseline models calculated direct healthcare costs over a ten-year timeframe, including the cost of the diagnostic process where appropriate but excluding indirect costs ascribable to caregiver time. For all scenarios involving an intervention we included the cost of a diagnostic assessment modelled on that recommended by current UK dementia guidelines [33]. This cost a total of £472.94 ($724; 2011 prices based on the Unit Costs of Health and Social Care 2011 [31] and the NHS National Schedule of Reference Costs Years 2011-12 [34]; see Additional file 1: Table S1) and comprised a GP visit including blood and biochemistry followed by either a CT or MRI scan (50% probability of each), and a memory clinic appointment comprising 60 minutes with a nurse, 45 minutes with a consultant psychiatrist, and with possible additional investigations, namely neuropsychology (10% of patients) and SPECT (5% of patients).

Quality of life scores were combined with survival rates to produce outcomes for quality-adjusted life years (QALYs) which were ascribed a value of £25,000 ($38,250). QALYs were derived based on a modification of a published algorithm [35] that describes QALYs in terms of MMSE score and independent versus institutional living. The full algorithm is as follows: utility = 0.408 + (0.01 MMSE) - (0.159 if institutionalized) - (0.004 NPI) + (0.051 if living with caregiver). We excluded the two final terms so the utility score was based only on MMSE and institutionalisation. The algorithm was used to ascertain utility for each MMSE score and differences in resulting QALYs in the intervention arm were ascribed based on MMSE score on a monthly basis.

### Modelling of interventions

Patients entering the early or standard intervention arms were assumed to receive a hypothetical intervention which varied with respect to the timing of the intervention, the immediate change in MMSE, and the effect of the intervention on disease course, which we modelled simply as a temporary halt in cognitive deterioration. Age-related mortality was applied from the point of early intervention throughout the rest of the model [36].

A. **Symptomatic treatment**

This model was based on the likely effects of using symptomatic treatments such as cholinesterase inhibitors. The baseline case compared the application of an intervention that immediately improved MMSE by one point, and where the beneficial effect wore off over 3 years, so that 3 years after intervention their MMSE score had converged with the original course. This intervention was applied at all points throughout the nine-year period and was compared against intervention at the end of the nine years, i.e. at actual time of diagnosis (standard intervention). The baseline assumption was that the effect size of intervention would be equal regardless of the time at which the intervention was applied, and costs and benefits were calculated initially using a ten year horizon. The impact of these two parameters was then assessed in sensitivity analyses. For all scenarios the average age at model start was 75 years.

B. **Disease modification**

There are various new potential disease-modifying therapies for AD-dementia under research [37]. This model assumed that a hypothetical intervention (based on possible future treatment developments rather than current available interventions), produced no acute change in MMSE but prevented further disease progression (change in MMSE score) for a given period, after which the natural course of decline would resume. This was compared against a model in which no such intervention was applied (‘no intervention’). The baseline case assumed that the intervention produced a 12 month delay in disease progression. Costs and benefits were calculated over a ten year horizon, and a mean age of 75 was assumed at model start. The impact of each of these parameters was then investigated in subsequent analyses.

## Results

### Symptomatic treatment

**Figure 1:**
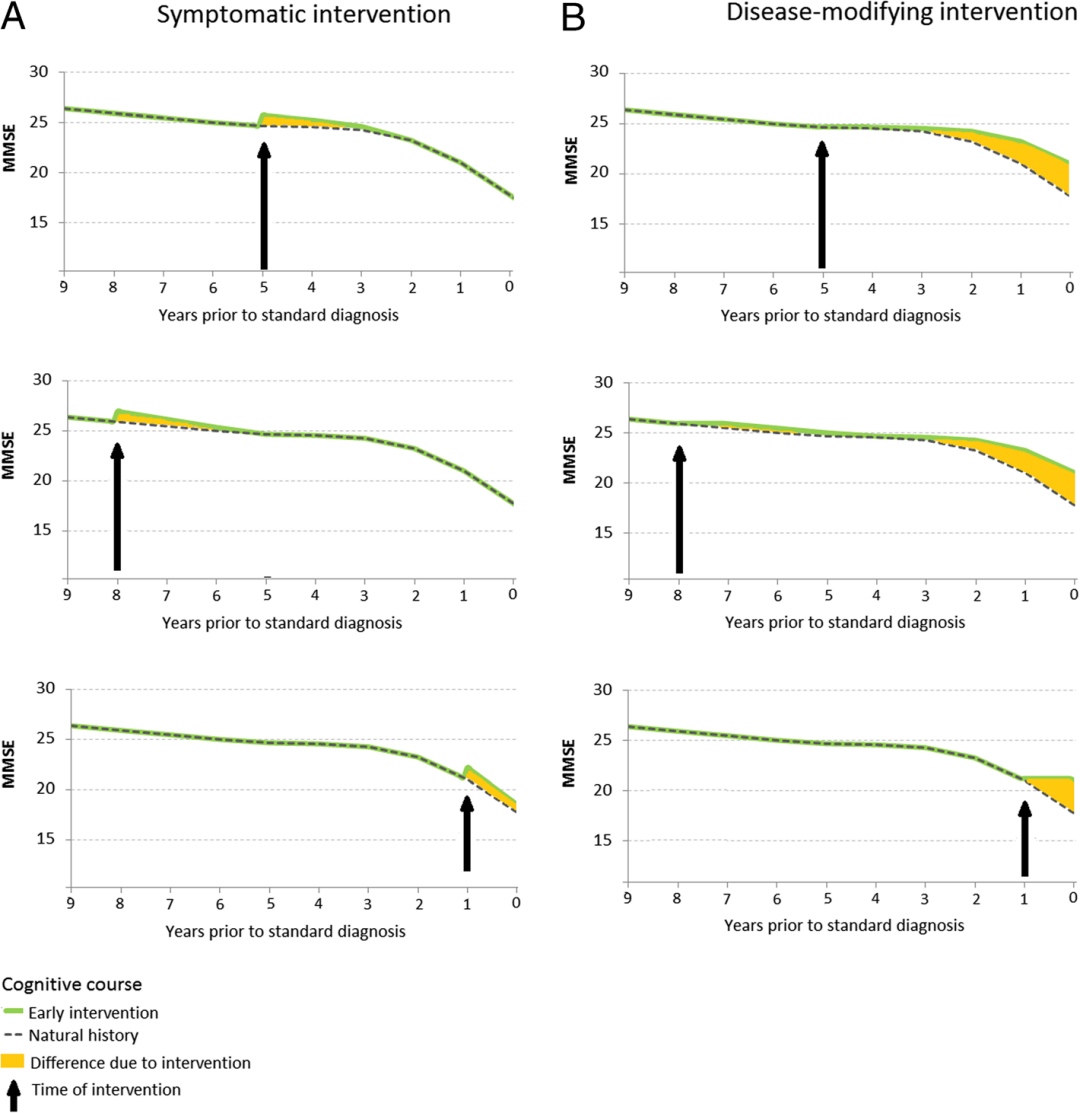
**Effects on cognitive course of symptomatic and disease-modifying interventions applied 5, 8 or 1 year prior to a standard diagnosis of Alzheimer’s disease.** Panel **A** symptomatic intervention at 5 (top), 8 (middle), and 1 (lower) years; Panel **B** disease-modifying intervention at 5 (top), 8 (middle), and 1 (lower) years.

**Figure 2:**
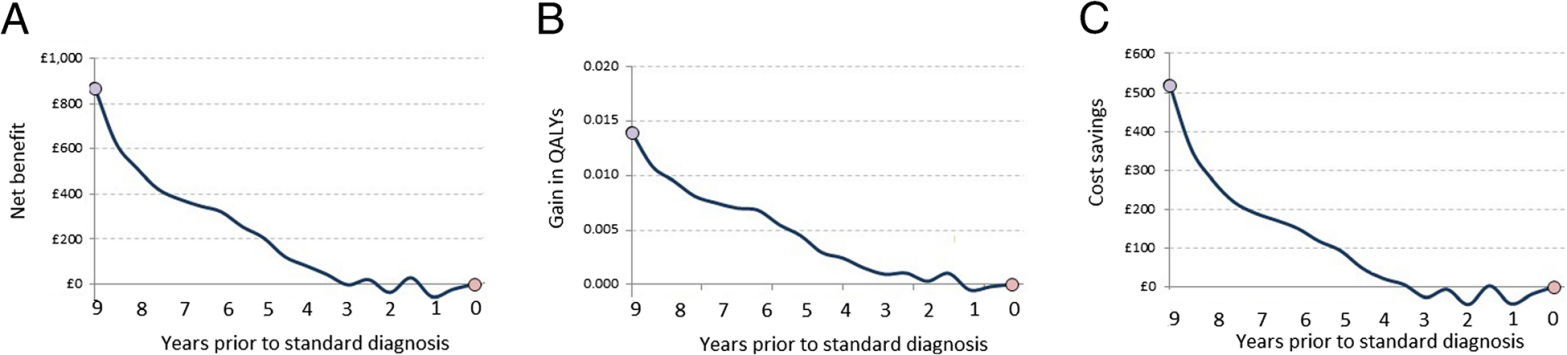
**Cost-effectiveness of early intervention with a modest symptomatic treatment in the nine years prior to standard detection and treatment.** Panel **A** net benefit; **B** Gain in quality-adjusted life years (QALYs); **C** incremental costs. Symptomatic effects = 1 MMSE point, efficacy reduces linearly over 3 years. Costs calculated over a 10-year horizon. Mean cohort age at entry 75 years.

We first evaluated the effect of a symptomatic improvement of one MMSE point, which declined over three years and was applied eight years before standard intervention. Figure 1A illustrates the difference in cognitive profile between early and standard interventions under this scenario. The economic benefits of intervention were greatest when treatment was applied at the earliest point in the model (i.e. 8 years prior to standard intervention; see Figure 2). However, it is important to point out that this models a hypothetical scenario (and, therefore, is not populated with data) using the assumption that MMSE was improved by one point. At 8 years prior to standard intervention, MMSE score is already high, reflecting MCI-AD, and, therefore, the assumption of one point improvement may overestimate that which be made at the higher end of the MMSE scale. The results should be interpreted with caution appropriate to a hypothetical model. At this timepoint, the overall net benefit of early intervention was £897 ($1372). There was no apparent economic benefit in intervening less than 3 years earlier than the standard, but in the period between 3 and 9 years prior to standard intervention, the net benefit of earlier intervention increased by around 17% per year.

Maximum economic differences between standard and early interventions, at -8 years, were: diagnostic cost £293 (standard; $448) versus £473 (early; $724); other healthcare costs £97,328 ($112,065) vs £96,608 ($147,810); gain in QALYs 4.246 vs 4.260 (Figure 2). Average diagnostic cost was less for standard intervention because some of those who would receive diagnosis under an early intervention protocol would not survive long enough to receive it under a standard intervention.

**Figure 3:**
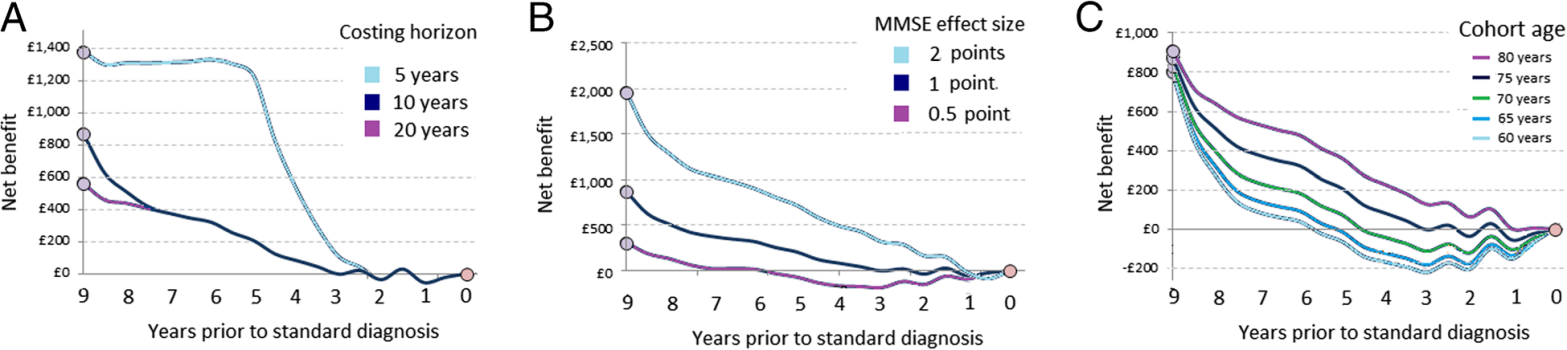
**Sensitivity of net benefits obtained under a symptomatic treatment model to costing horizon and treatment effect size.** Panel **A**: results when costing over 5, 10 and 20 years; Panel **B**: result of doubling or halving of treatment effect on MMSE score; Panel **C**: effect of mean cohort age at model start.

To test the sensitivity of this model to the assumptions regarding costing horizon and treatment efficacy we repeated the model while varying these assumptions (Figure 3). Reducing the costing horizon from 10 to 5 years after early intervention greatly increased the incremental benefits of earlier intervention, because fewer had the opportunity to benefit from standard detection. Conversely, extending the costing horizon to 20 years had relatively little effect (Figure 3A). Doubling or halving the treatment effect on MMSE produced corresponding changes in net benefit that were greater in absolute magnitude the earlier the treatment was applied (Figure 3B). For a treatment that improved cognition by only half an MMSE point, no net benefit was seen if applied less than 7 years before standard intervention. Net benefits were greater, and the benefits of early intervention exacerbated, among older cohorts (Figure 3C), where mortality effects meant that fewer would have lived to receive the benefit of standard intervention.

### Disease-modification

**Figure 4:**
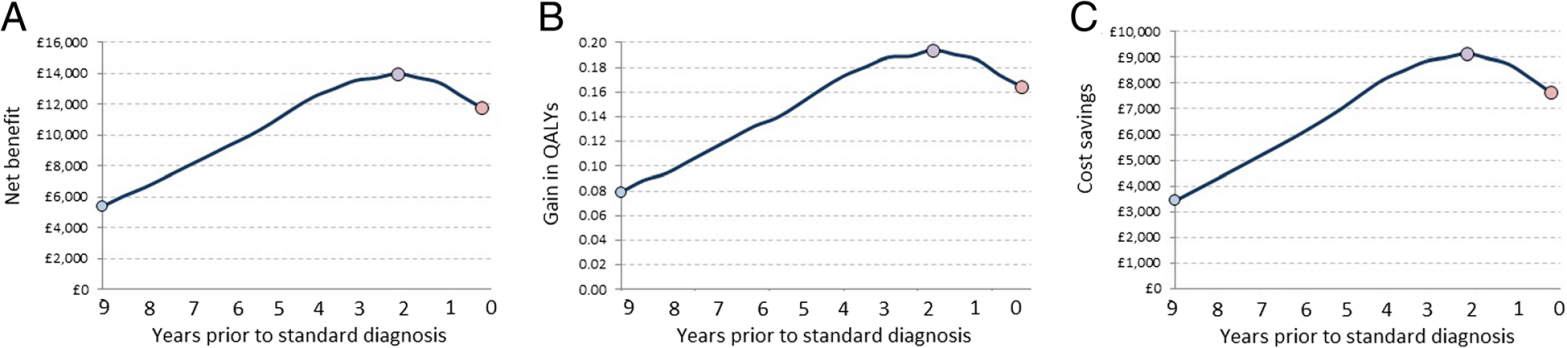
**Cost-effectiveness of early intervention with a hypothetical disease-modifying treatment in the nine years prior to standard detection and treatment.** Panel **A** net benefits; **B** Gain in quality-adjusted life years (QALYs); **C** incremental costs. Treatment effect is a 12-month halt in cognitive deterioration. Costs calculated over a 10-year horizon. Mean cohort age at entry 75 years. Panel **A**, net benefits; Panel **B**, gain in quality-adjusted life years (QALYs); Panel **C** cost savings. Treatment effect is a 12-month halt in cognitive deterioration. Costs calculated over a 10-year horizon. Mean cohort age at entry 75 years.

We then compared the effect of a hypothetical disease-modifying intervention which halted cognitive deterioration, against no such intervention. In the baseline model, treatment halted deterioration for 12 months, after which the natural progress of cognitive decline resumed. Figure 1B illustrates the effect that this intervention has on the cognitive course. The benefits were greatest when the intervention occurred around two years prior to standard intervention, at the point where MMSE deterioration is greatest. Figure 4 shows the incremental difference in healthcare costs, the gain in QALYs and the net benefit for intervening across the nine-year timeframe versus no intervention. The comparative costs for intervening at the optimal timepoint, versus no such intervention, were: diagnostic cost £0 (no intervention) versus £472 (intervention) ($722); other healthcare costs £121,790 ($186,339) vs £112,170 ($171,620); gain in QALYs 2.454 vs 2.648, for an overall net benefit of intervention of £13,996 ($21,414).

**Figure 5:**
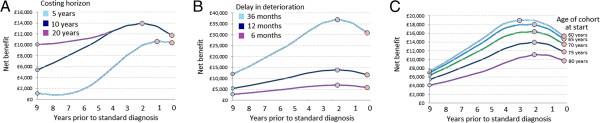
**Sensitivity of net benefits obtained through a hypothetical disease-modifying treatment to costing horizon, treatment effect and age of cohort.** Panel **A**: results when costing over 5, 10 and 20 years; Panel **B**: result of increased and decreased treatment effect, as expressed in months delay in deterioration in MMSE score; Panel **C**: effect of mean cohort age at model start.

To test sensitivity to the assumptions regarding costing horizon, treatment efficacy and age of cohort we again repeated the model while varying each of these parameters (Figure 5). All three considerably affected the magnitude of net benefit observed. In contrast to the symptomatic model, net benefits were greater over longer costing horizons (Figure 5A) and time of intervention had a greater impact on net benefit when considering the shortest costing horizon (5 years). Treatment efficacy, modelled as length of delay in cognitive deterioration had a particularly large effect on cost-effectiveness (Figure 5B). For example, a treatment that delayed deterioration by 36 months and that was applied 2 years prior to standard intervention provided a net benefit of £37,098 ($56,760). Net benefits were greater, and the effect of early intervention exacerbated, among younger cohorts, where mortality over the post-diagnostic period would be lower.

### Comparison of the effect of timing of intervention for symptomatic versus disease-modifying treatments

The two intervention scenarios had very different effects on the overall course of cognitive function. For the symptomatic treatment, the benefits of intervention were inherently time-limited and roughly equal in magnitude with respect to the overall effect on cognitive course (area under the curve in Figure 1A). In contrast, the nature of the disease-modifying intervention was such that the immediate effect of the intervention was driven by the nature of the cognitive decline function at the time of intervention, and the majority of the effect on cognition was seen relatively late in the decline curve (Figure 1B). In absolute terms, the maximum net benefit of the disease modifying intervention modelled here was fifteen times greater than for the modelled symptomatic treatment.

## Discussion

In this study we utilised the known natural history of cognitive decline in Alzheimer’s disease to estimate the health economic impact of earlier intervention. We modelled two hypothetical scenarios; one a symptomatic drug with effects similar to those seen for cholinesterase inhibitors, the other a disease-modifying intervention that delayed cognitive deterioration for a period of time. In both cases, results showed that optimally timing an intervention could significantly affect its economic benefits. For the symptomatic treatment, the economic benefits were greater the earlier the intervention was applied; in contrast the disease-modifying intervention was most cost-effective when applied relatively late in the course.

These differences in optimal timing between symptomatic and disease-modifying models can be explained by the effects of mortality on post-diagnostic healthcare costs and by the changing rate of cognitive decline throughout the disease course. For the symptomatic intervention, treatment benefits are immediate and decrease over a fixed time period. Intervention is consequently most effective when applied at the earliest possible timeframe, so that the greatest number of individuals benefit from the intervention before any loss to mortality. However the effect of this intervention is relatively small because it is inherently time-limited. In contrast, in the disease-modifying model, while there is no immediate impact, the effect of intervention is to permanently shift the curve of cognitive decline. As a result, the effect on both healthcare costs and QALYs is considerably larger. Under this scenario, intervention was most economically beneficial when applied around two years prior to the time of standard diagnosis. This is the period during which MMSE decline is most rapid: intervening either earlier or later than this reduces the costs saved by delaying cognitive decline.

Other health economic models of disease modifying treatments have also estimated higher costs with DMT over time, due to additional costs of DMT and the increased survival with DMT [38].

Of the two interventions, the results from the symptomatic model are more immediately applicable because moderately effective symptomatic treatments are already available in the UK for patients with mild to severe AD -dementia (MMSE of 26 or below). In this cohort, individuals already had a mean MMSE score indicating AD-dementia treatment up-to nine years before diagnosis, so these results are in one sense supporting not ‘early’, but rather ‘on time’ intervention. While we have not included treatment costs in the model, generic AD-dementia drugs now cost just pennies per day. These results therefore support previous analyses demonstrating the cost-effectiveness of intervention and treatment using symptomatic treatments in the NHS, and extend this by arguing that intervention will be more cost-effective when applied earlier than currently occurs for the majority of patients.

The disease-modifying results should not be interpreted to mean that we can wait until frank dementia before intervening. On the contrary, to maximise the cost-effectiveness of a disease-modifying strategy, the benefits of such a treatment must be being felt before patients hit the period of rapid decline that precedes a diagnosis in this cohort. In order to prevent this precipitous decline it will, at a minimum, be necessary to detect and closely monitor individuals with milder cognitive impairment so that intervention can be certain to occur prior to that point of inflection. In all likelihood, the mode of action of such an intervention, be it a lifestyle prevention strategy or anti-amyloid immunisation, would probably need intervention to start many years or decades earlier in order to have an impact on cognitive course.

Our model included full direct health and social care costs and the cost of the diagnostic procedure but excluded any costs associated with informal caregiver time [39,40], and, importantly, the cost of the intervention itself. This might range from a few pounds per month for a generic drug such as a cholinesterase inhibitor to many thousands for a future biological therapy. Alternatively, modification of disease course and its impact, could potentially be obtained from non-pharmacological strategies such as lifestyle intervention which might be required decades earlier (i.e. as a primary prevention strategy) in order to prevent the disease process from occurring [41]. Although excluding the cost of informal care narrows the viewpoint somewhat, excluding these costs takes a conservative approach. Including caregiver costs would cause the results to be more favourable towards early detection. A sensitivity analysis demonstrated that the inclusion of a cost associated with the intervention has a linear impact on the model’s findings. That is, if the cost of the intervention is £50, then the total cost savings associated with early detection will be reduced by £50. If the cost of the intervention is £200, the cost savings are reduced by £200.

We did not include in our models a specific cost of treatment, so these results provide a framework for estimating the likely bearable costs to the UK health system for a sufficiently effective anti-dementia intervention. The results show that cost-effectiveness might be achieved with either a cheap symptomatic treatment or a more expensive disease-modifying one. The treatment effects modelled here were purposefully modest and reflect the relatively small effects achieved thus far in both approved and developing compounds. While both the healthcare costs and QALY values used here are taken from the UK system, the principles are likely to apply widely within European and North American countries, where under-diagnosis is common [17-19] and where any intervention which improved the function of AD-dementia patients would be expected to have a proportionally similar effect on healthcare costs [42].

An important limitation of the model is the assumption that the efficacy of the intervention does not depend on when it is applied, either with respect to illness severity, or to cohort age. At present little data exists addressing this for existing treatments, although for future anti-amyloid therapies intervention is likely to be more effective when applied earlier in disease course. Age at treatment start is particularly important because at the older ages, mortality plays an increasing role in exaggerating the economic differences between early and standard intervention. In the absence of published data, we ignored the effect of treatment on survival, whilst in reality disease-modifying interventions, in particular, would be expected to play a role in reducing mortality. All of these limitations are conservative in nature and would lead, if anything, to an underestimation by the present model of the benefits of earlier intervention.

The model also does not take into account the possible effect of false positives. As diagnoses of AD-dementia shifts to earlier disease states the difference between pathological symptoms and normal age-related cognitive decline is likely to be subtle, with the potential for greater diagnostic uncertainty, leading to an increased risk of a false positive diagnosis (Albert, 2011). Due to a lack of data this was not included in the model; however the likely impact of including this would be that in earlier diagnosis there are more false positives, leading to treatment costs increasing, while no extra benefit is incurred. This should be considered in future studies and when translating our analyses.

## Conclusions

In summary, we demonstrate that for both symptomatic and disease-modifying interventions, timing is crucial in determining the economic benefits of a treatment, even leaving aside the issue of treatment efficacy. Early intervention is clearly indicated for current symptomatic treatments, which are likely to be most cost-effective when applied as early as it is possible to diagnose AD-dementia. For a disease-modifying intervention, maximal cost-effectiveness would be achieved by intervening early enough to anticipate the point at which cognition begins a rapid decline. Taken together, these results suggest that cost-effective early detection and intervention should be an achievable goal in earlier stages of AD (MCI-AD), and that a range of different intervention effects and costs might be manageable or even beneficial in terms of overall healthcare costs. Public policy including screening, diagnostic and prescribing guidelines should aim to reflect the optimal cognitive stage for cost-effective intervention, which will be several years prior to current standard practice.

## Competing interests

JB & AB are employees of, and shareholders in, Cambridge Cognition, a company which produces cognitive tests for use in research and healthcare. LL and MT report no potential conflicts.

## Authors’ contributions

JB and AB conceived the idea for the study. LL and MT conceived and built the economic model. JB led, and all authors contributed to, the drafting of the manuscript. All authors read and approved the final manuscript.

## Pre-publication history

The pre-publication history for this paper can be accessed here:

http://www.biomedcentral.com/1471-2377/14/101/prepub

## Supplementary Material

Additional file 1: Table S1 Costs of standard diagnostic process, based on the Personal Social Services Research Unit report ‘Unit Costs of Health and Social Care 2011’ and the ‘National Schedule of Reference Costs Years 2011–12, NHS Trusts and NHS Foundation Trusts’. Click here for file
