# Supplementary material for: Early intervention in Alzheimer’s disease: a health economic study of the effects of diagnostic timing
Source: BMC Neurol. 2014 May 7;14:101. doi: 10.1186/1471-2377-14-101 (PMC4032565; doi:10.1186/1471-2377-14-101)
Supplement: Additional file 1: Table S1 — Costs of standard diagnostic process, based on the Personal Social Services Research Unit report ‘Unit Costs of Health and Social Care 2011’ and the ‘National Schedule of Reference Costs Years 2011–12, NHS Trusts and NHS Foundation Trusts’. [file 1471-2377-14-101-S1.doc]

| Cost centre | Specifications | Unit cost | Proportion who receive procedure | Mean cost per patient |
| --- | --- | --- | --- | --- |
| MRI scan | One area no contrast | £145 | 50% | £72.50 |
| CT scan | One area no contrast | £92 | 50% | £46 |
| SPECT scan | Nuclear med cat 3 - medium cost isotope | £238 | 5% | £11.90 |
| Haematology | Unit | £3.09 | 100% | £3.09 |
| Biochemistry | Unit | £1.23 | 100% | £1.23 |
| Other pathology | Unit | £6.47 | 100% | £6.47 |
| GP appointment | Per 17.2 minute clinic visit | £53 | 100% | £53 |
| Nurse appointment | Per contact hour mental health nurse | £65 | 100% | £65 |
| Neuropsychology | Per contact hour clinical psychologist | £135 | 10% | £13.50 |
| Consultant psychiatrist | Per 45 minutes, patient-related | £200.25 | 100% | £200.25 |
|  |  |  |  |  |
| **Total costs** |  |  |  | **£472.94** |
